# Accessibility of the three-year comprehensive prevention and control of brucellosis in Ningxia: a mathematical modeling study

**DOI:** 10.1186/s12879-023-08270-4

**Published:** 2023-05-05

**Authors:** Wei Gong, Peng Sun, Changsheng Zhai, Jing Yuan, Yaogeng Chen, Qun Chen, Yu Zhao

**Affiliations:** 1 School of Science, Ningxia Medical University, 750001 Yinchuan, China; 2 Science and Technology Center, Ningxia Medical University, 750001 Yinchuan, China; 3 School of Mathematics and Computer Science, Ningxia Normal University, 756000 Guyuan, China; 4 School of Public Health and Management, Ningxia Medical University, 750001 Yinchuan, China; 5 Key Laboratory of Environmental Factors and Chronic Disease Control, 750001 Yinchuan, China

**Keywords:** Brucellosis, Dynamical modeling, Control strategy, Basic reproduction number, Accessibility evaluation

## Abstract

**Background:**

Brucellosis is a chronic zoonotic disease, and Ningxia is one of the high prevalence regions in China. To mitigate the spread of brucellosis, the government of Ningxia has implemented a comprehensive prevention and control plan (2022-2024). It is meaningful to quantitatively evaluate the accessibility of this strategy.

**Methods:**

Based on the transmission characteristics of brucellosis in Ningxia, we propose a dynamical model of sheep-human-environment, which coupling with the stage structure of sheep and indirect environmental transmission. We first calculate the basic reproduction number $R_0$ and use the model to fit the data of human brucellosis. Then, three widely applied control strategies of brucellosis in Ningxia, that is, slaughtering of sicked sheep, health education to high risk practitioners, and immunization of adult sheep, are evaluated.

**Results:**

The basic reproduction number is calculated as $R_{0}=1.47$, indicating that human brucellosis will persist. The model has a good alignment with the human brucellosis data. The quantitative accessibility evaluation results show that current brucellosis control strategy may not reach the goal on time. “Ningxia Brucellosis Prevention and Control Special Three-Year Action Implementation Plan (2022-2024)” will be achieved in 2024 when increasing slaughtering rate $\gamma$ by 30$\%$, increasing health education to reduce $\beta _{h}$ to 50$\%$, and an increase of immunization rate of adult sheep $\theta$ by 40$\%$.

**Conclusion:**

The results demonstrate that the comprehensive control measures are the most effective for brucellosis control, and it is necessary to further strengthen the multi-sectoral joint mechanism and adopt integrated measures to prevention and control brucellosis. These results can provide a reliable quantitative basis for further optimizing the prevention and control strategy of brucellosis in Ningxia.

**Supplementary Information:**

The online version contains supplementary material available at 10.1186/s12879-023-08270-4.

## Introduction

Brucellosis is one kind of infectious diseases caused by bacteria of the genus brucella that is common to mammals host such as cattle, sheep, pigs [1, 2]. Human brucellosis is usually transmitted by the following ways: 1) the infected females eject placenta and foetuses into the environment during abortion and lambing periods [3], the main transmission sources of human brucellosis is the susceptible infected the livestock and brucella in the exposed environment, and there is no recorded transmission of the infection between humans [4]. 2) Bacteria can float in the air as aerosols after infected, so that people can breathe into the respiratory tract and become infected [5, 6]. Brucellosis spreads first among infected animals, causing infection or disease, and then to the humans. It has similar clinical features to many infectious diseases, such as fever, weak, joint pain, lymphadenectasis etc. [7]. In addition, brucellosis mainly affects reproduction and fertility to animals, reducing the survival rate of newborns [8]. It has spread in more than 170 countries in the world, with higher numbers of severe cases in Central Asia, the Middle East, Outer Mongolia and China [9]. Human and animal cases have been reported in 31 provinces (municipalities and autonomous regions) in China. Epidemiological studies reported that [10, 11] human brucellosis has a significant spatio-temporal heterogeneity, the high aggregation area mainly occur in the pastoral areas of the northern provinces, and the incidence varies from $<0.01$ to $>200$ per 100,000 population. Thus, how to effectively perform the prevention and control of brucellosis in high risk regions is still an important and meaningful public health issue.

**Fig. 1:**
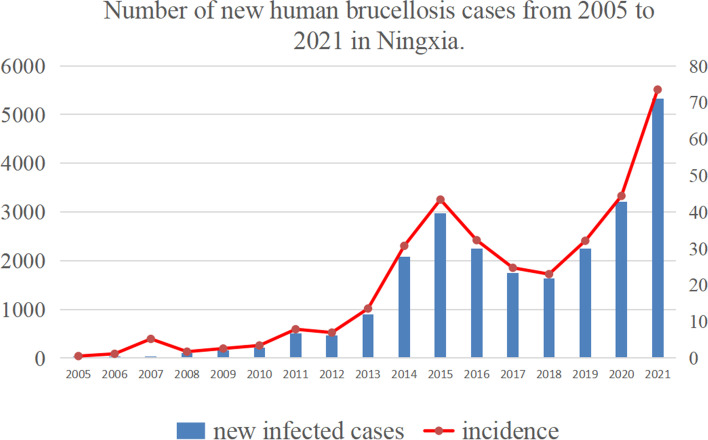
Number of new human brucellosis cases from 2005 to 2021 in Ningxia

Ningxia is located in the northwest of China and also a high prevalence area for brucellosis. In the 1980s, Ningxia was officially classified as a “brucellosis control area”, and in 2004, human cases of brucellosis resurfaced in Ningxia, the epidemic continued to spread, gradually expanding to 22 counties (cities and districts) by 2013. As shown in Fig. 1, a total of 23,867 human cases of brucellosis were reported in Ningxia from 2005 to 2021, and the incidence rising from 0.02/100,000 in 2004 to 43.39/100,000 in 2015, then decreasing to 22.97/100,000 in 2018, and rebound to history high value 73.5/100,000 in 2021. Thus, the situation of brucellosis prevention and control in Ningxia is severe and urgent. In recent years, with the implementation of the rural revitalization strategy, the amount of livestock breeding is increasing, the circulation of animals and their products are frequent. Sheep breeding industry as an important part of the economic industry in Ningxia, the reemerging brucellosis poses a heavy economic burden. In order to mitigate the influence of brucellosis on the sheep breeding industry, and ensure the healthy development of the breeding industry and protect people’s health, the government of Ningxia implemented “Ningxia Brucellosis Prevention and Control Special Three-Year Action Implementation Plan (2022-2024)”. However, the effect the three-year comprehensive prevention and control of brucellosis in Ningxia is still unclear, thus, assessing the accessibility of the three-year comprehensive prevention and control of brucellosis in Ningxia may provide a reliable reference for the policy formulation of brucellosis prevention and control strategy.

Mathematical modeling is a powerful tool for analyzing the complexity of transmission mechanisms and epidemiological features of infectious diseases and can suggest new approaches for the prevention and control of future epidemics [12–19]. Dynamic models can provide valuable insights into the transmission characteristics of brucellosis and have yielded positive results [20–23]. For example, Li et al. [24] proposed a multi-herd SEIRV dynamic model of bidirectional mixed cross-infection in cattle and sheep, involving both young and adult sheep, and studied the effect of cross-infection in mixed feeding on the transmission of brucellosis. It should be noted that infectious sheep can shed brucella into the environment, and brucella can be harvested by susceptible individuals that become infected [25]. The indirect transmission (environmental transmission) plays a relatively small role on the transmission of brucellosis, however, it should not be ignored. Taking the impact of brucellosis in the environment into consideration, $A\dot{i}nseba$ et al. [26] proposed a dynamic SIC model for the transmission of sheep brucellosis through direct and indirect environmental infection ways and discussed the indirect environmental infection on the brucellosis transmission dynamics. Hou et al. [27] proposed a dynamic model of sheep-human transmission involving brucellosis in sheep populations, human populations, and the environment in combination with the characteristics of brucellosis infection in the Inner Mongolia Autonomous Region. Qin et al. [28] studied the effect of sheep migration on the spread of the disease and developed a patch dynamics model. The basic reproduction number, disease-free equilibrium and positive equilibrium of the model were discussed. Studies have shown that appropriate protective measures play an important role in curbing the spread of infectious diseases [29]. Recently, Ma et al. [30] studied the effect of different control measures on the spread of brucellosis in Jilin Province, China, using a discrete-time human-sheep coupling mathematical model established by the inverse Euler method. The results indicated that expanding the frequency of disinfection and strengthening the effect of publicity and education could shorten the epidemic time and final size of brucellosis in Jilin Province. Related results can see [31–33] for more details.

Most of the human brucellosis cases are infected by sheep-type brucella, accounting for 84.5 $\%$ of the total cases in China [34], and sheep brucellosis is also the main infection source of human brucellosis in Ningxia [11]. Related studies reported that [35, 36] the degree of sheep brucella infection may positively associated with the increase of age and sexual maturity. Generally, lambs (from birth to 4 months of age) has a more stronger resistant to brucella than that of adult sheep (over 5 months). Brucellosis in sheep has a significant stage structure, with a low incidence of brucellosis in lambs and a high susceptibility in adult sheep. In addition, immunization sheep is one of the effective methods to prevent and control the spread of sheep, such as brucellosis vaccine S2, live brucellosis vaccine for sheep (M5 strain), etc. Then, immunodetection of the adult sheep is used to detected and innocent treatment the brucellosis positive sheep [34, 37]. Thus, considering the stage structures of sheep is benefit to deeply understanding the prevention and control strategy of brucellosis.

Motivated by above, based on the transmission characteristics of brucellosis in Ningxia, we propose a dynamical model of sheep-human-environment, which coupling with the stage structure of sheep and indirect environmental transmission. In theory, we obtain the basic reproduction number $R_0$ of the model, which determines the dynamics: if $R_0<1$, the disease-free equilibrium E^0^of model is globally asymptotically stable, while $R_0<1$, the endemic equilibrium $E^*$ of model is globally asymptotically stable. In practice, we first use the model to fit the incidence of human brucellosis, and the fitted results are well accordance with the data of human brucellosis in Ningxia. Then, we further quantitatively evaluate the accessibility of “Ningxia Brucellosis Prevention and Control Special Three-Year Action Implementation Plan (2022-2024)” by slaughtering the sicked sheep, improving the health education to breeding practitioners and increasing the immunization of adult sheep.

## Materials and methods

### Data sources

The number of reported brucellosis cases in Ningxia from 2005 to 2021 was obtained from the Infectious Disease Reporting Information Management System of Ningxia Center for Disease Control and Prevention (http://nxcdc.org/article/1940), and the population data was obtained from Ningxia Statistical Yearbook (http://nxdata.com.cn/publish.htm?cn=G01).

### Model formulation

**Fig. 2:**
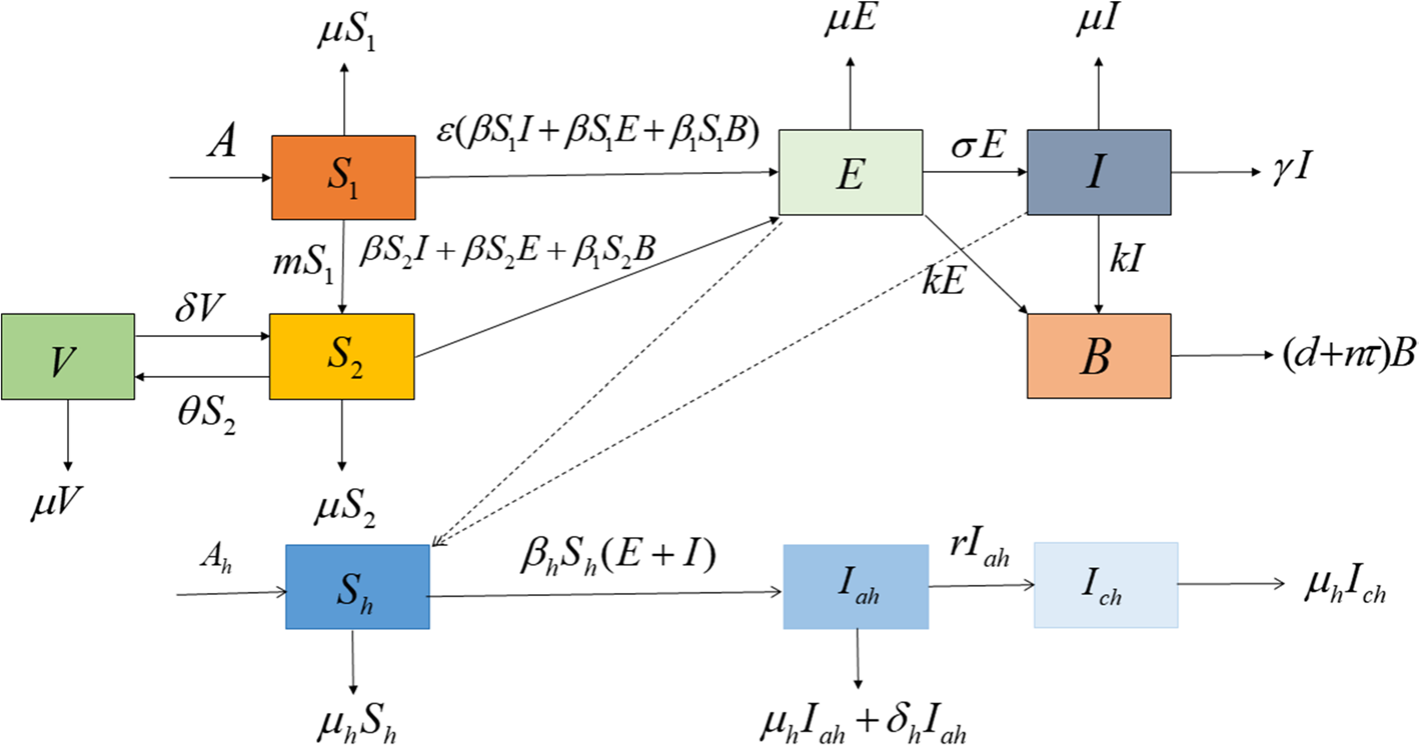
The transmission diagram of brucellosis

In this subsection, according to the transmission characteristics of brucellosis in Ningxia, we propose a sheep-human-environment coupling mathematical model, which considering that the two-stage structure (young (1$\sim$4 months) and adult (over 5 months). The transmission diagram of brucellosis is shown in Fig. 2. For sheep population, we classify sheep into five compartments: susceptible lambs $S_{1}$, susceptible adult sheep $S_{2}$, latent sheep *E*, infected sheep *I*  and immunodetection sheep *V*.  *B* represents brucella in the environment. For human population, the clinical symptoms do not have obvious characteristics, it is assumed that once infected with brucellosis, it is generally found to be an acute patient, thus, we divide into susceptible $S_{h},$ acute infectious $I_{ah},$ and chronic infectious $I_{ch}.$

We assume that:

1) The parameters of the model are all non-negative;

2) Newborn sheep are considered susceptible;

3) The infection rate of infected human is the same after infection;

4) The birth rate of sheep is related to both adult and immune species;

5) The flock is a constant input and only enters the susceptible lamb compartment;

6) Take timely slaughtering measures for sicked sheep;

7) The natural mortality of the same population is the same at different times.

Based on the above assumptions, a staged-structure sheep-human-environment dynamical mathematical model is given as follows:

1 $$\begin{aligned} \left\{ \begin{array}{l} \frac{\textrm{d}S_{1}}{\textrm{d}t}=A+\alpha (S_{2}+V)-(\mu +m)S_{1}-\varepsilon S_{1}(\beta I+\beta E+\beta _{1}B),\\ \frac{\textrm{d}S_{2}}{\textrm{d}t}=mS_{1}-\theta S_{2}-\mu S_{2}+\delta V-(\beta S_{2}I+\beta S_{2}E+\beta _{1}S_{2}B),\\ \frac{\textrm{d}E}{\textrm{d}t}=\varepsilon S_{1}(\beta I+\beta E+\beta _{1}B)+(\beta S_{2}I+\beta S_{2}E+\beta _{1}S_{2}B)-(\mu +\sigma )E,\\ \frac{\textrm{d}I}{\textrm{d}t}=\sigma E-(\mu +\gamma )I,\\ \frac{\textrm{d}V}{\textrm{d}t}=\theta S_{2}-(\mu +\delta )V,\\ \frac{\textrm{d}B}{\textrm{d}t}=k(E+I)-(d+n\tau )B,\\ \frac{\textrm{d}S_{h}}{\textrm{d}t}=A_{h}-\beta _{h}S_{h}(E+I)-\mu _{h}S_{h},\\ \frac{\textrm{d}I_{ah}}{\textrm{d}t}=\beta _{h}S_{h}(E+I)-(\mu _{h}+\delta _{h}+r)I_{ah},\\ \frac{\textrm{d}I_{ch}}{\textrm{d}t}=rI_{ah}-\mu _{h}I_{ch}. \end{array}\right. \end{aligned}$$

The meaning of each parameter in system (1) is listed in Table 1. In model (1), the first eight equations are independent of $I_{ch},$ so we only need to consider model (2) for theoretical analysis.

2 $$\begin{aligned} \left\{ \begin{array}{l} \frac{\textrm{d}S_{1}}{\textrm{d}t}=A+\alpha (S_{2}+V)-(\mu +m)S_{1}-\varepsilon S_{1}(\beta I+\beta E+\beta _{1}B),\\ \frac{\textrm{d}S_{2}}{\textrm{d}t}=mS_{1}-\theta S_{2}-\mu S_{2}+\delta V-(\beta S_{2}I+\beta S_{2}E+\beta _{1}S_{2}B),\\ \frac{\textrm{d}E}{\textrm{d}t}=\varepsilon S_{1}(\beta I+\beta E+\beta _{1}B)+(\beta S_{2}I+\beta S_{2}E+\beta _{1}S_{2}B)-(\mu +\sigma )E,\\ \frac{\textrm{d}I}{\textrm{d}t}=\sigma E-(\mu +\gamma )I,\\ \frac{\textrm{d}V}{\textrm{d}t}=\theta S_{2}-(\mu +\delta )V,\\ \frac{\textrm{d}B}{\textrm{d}t}=k(E+I)-(d+n\tau )B,\\ \frac{\textrm{d}S_{h}}{\textrm{d}t}=A_{h}-\beta _{h}S_{h}(E+I)-\mu _{h}S_{h},\\ \frac{\textrm{d}I_{ah}}{\textrm{d}t}=\beta _{h}S_{h}(E+I)-(\mu _{h}+\delta _{h}+r)I_{ah},\\ \end{array}\right. \end{aligned}$$

with the initial conditions

3 $$\begin{aligned} \left\{ \begin{array}{l} S_{1}(\theta )=\eta _{0}(\theta ),S_{2}(\theta )=\eta _{1}(\theta ),E=\eta _{2}(\theta ),I=\eta _{3}(\theta ),V=\eta _{4}(\theta ),B=\eta _{5}(\theta ),S_{h}=\eta _{6}(\theta ),\\ I_{ah}=\eta _{7}(\theta ),\eta _{i}(\theta )\ge 0,\eta _{i}(0)>0,\eta _{i}\in \mathbb {C}(R_{+}),i=0,1,2,3,4,5,6,7, \end{array}\right. \end{aligned}$$

where $$R_{+}=\{x\in R^{+}_{8}: x\ge 0\},$$ and $$\mu >\alpha .$$ 

### Accessibility evaluation

After the reported incidence of human brucellosis in Ningxia reached a historical peak in 2015 (43.39/100,000), the government of Ningxia promptly implemented “Ningxia inter-animal brucellosis spring and autumn immunization program(2016-2018)”, so that the inter-animal epidemic was alleviated, and the reported incidence of human brucellosis decreased for three consecutive years. The incidence decreased in the subsequent three years, but Ningxia still ranked among the top three in China for three consecutive years in the national ranking of reported incidence. In view of the serious situation of the human brucellosis in Ningxia, the government of Ningxia has implemented “Ningxia Brucellosis Prevention and Control Special Three-year Action Implementation Plan (2022-2024)”, which aims to reducing the incidence rate of human brucellosis by more than 60$\%$ per year by 2024.

According the epidemiological history and transmission route of brucellosis, we proposed a coupled sheep-human-environment mathematical model with stage structure. In order to further explore effective measures to prevent and control the epidemic of human brucellosis, the surveillance data of human brucellosis in Ningxia from 2005 to 2021 was used to validate the fit of goodness of the dynamical model, and then the effect of slaughtering of sicked sheep, health education to high risk practitioners, immunization of adult sheep on the brucellosis transmission are evaluated respectively. The evaluation of the accessibility was carried out by numerical simulation and sensitivity analysis.

### Theoretical results of model (2)

In theoretical epidemiology, one of the most important indicators to assess the risk of an infectious disease is the basic reproduction number (denoted $R_{0}$), which can be considered as the average number of cases produced by a case during its period of infection [38]. The asymptotic dynamic behavior of an infectious disease implies that the disease will die or persist in the future and can be reflected by the steady state. Therefore, we first provide some mathematical analysis results of model (2).

**Theorem 1.** The solution of model (2) with the initial value (3) is positive and bounded, and there is an invariant set $\Gamma$: 

$$\begin{aligned} \Gamma =\{(S_{1},S_{2},E,I,V,B,S_{h},I_{ah})\in R^{+}_{8}:0\le S_{1}+S_{2}+E+I+V\le \frac{A}{\mu -\alpha }, \end{aligned}$$

$$\begin{aligned} S_{h}+I_{ah}\le \frac{A_{h}}{\mu _{h}}, B\le \frac{kA}{(\mu -\alpha )(d+n\tau )}\}. \end{aligned}$$

Making use of the next generation matrix (see [39]), we obtain the basic reproduction number of model (2) as follows:

4 $$\begin{aligned} R_{0}=\underbrace{\frac{\varepsilon \beta S_1^0(\mu +\gamma +\sigma )}{(\mu +\sigma )(\mu +\gamma )}}_{\text {infected by sicked lambs}}+\underbrace{\frac{\sigma \beta S_{2}^{0}(\mu +\gamma +\sigma )}{(\mu +\sigma )(\mu +\gamma )}}_{\text {infected by sicked adult sheep}} +\underbrace{\frac{k(\mu +\sigma +\gamma )(\varepsilon \beta _{1} S_{1}^{0}+\beta _{1}S_{2}^{0})}{(\mu +\sigma )(\mu +\gamma )(d+n\tau )}}_{\text {infected from environment}}. \end{aligned}$$

**Proof.** The proof of Theorem 1 is given in A.1. of the supplementary material.

**Remark 1.** $R_{0}$ consists of three parts representing the infection of brucellosis from three sources, e.g. the first part represents infection caused by the sicked lambs, the second part represents infection caused by the sicked adult sheep, and the third part represents infection from the polluted environment.

#### Equilibrium

For system (2), there are two equilibria, that is,

1) disease-free equilibrium  $E^{0}=(S_{1}^{0},S_{2}^{0},0,0,V^{0},0,S_{h}^{0},0),$ where

$$\begin{aligned} S_{1}^{0}=\frac{A\mu }{\mu ^{2}+\mu m-\alpha m},S_{2}^{0}=\frac{Am(\mu +\delta )}{(\mu ^{2}+\mu m-\alpha m)(\mu +\delta +\theta )}, \end{aligned}$$

$$\begin{aligned} V^{0}=\frac{Am\theta }{(\mu ^{2}+\mu m-\alpha m)(\mu +\delta +\theta )},S_{h}^{0}=\frac{A_{h}}{\mu _{h}}, \end{aligned}$$

and 2) endemic equilibrium $E^{*}=(S_{1}^{*},S_{2}^{*},E^{*},I^{*},V^{*},B^{*},S_{h}^{*},I_{ah}^{*}),$ where

$$\begin{aligned} S_{1}^{*}=\frac{A[(\mu +\delta )(I^{*}H+\theta +\mu )-\delta \theta ]}{(I^{*}H\varepsilon +\mu +m)[(\mu +\delta )(I^{*}H+\theta +\mu )-\delta \theta ]-\alpha m(\mu +\delta +\theta )}, \end{aligned}$$

$$\begin{aligned} S_{2}^{*}=\frac{Am(\mu +\delta )}{(I^{*}H\varepsilon +\mu +m)[(\mu +\delta )(I^{*}H+\theta +\mu )-\delta \theta ]-\alpha m(\mu +\delta +\theta )}, \end{aligned}$$

$$\begin{aligned} E^{*}=\frac{(\mu +\gamma )I^{*}}{\sigma },I^{*}=\frac{\sigma E^{*}}{\mu +\gamma }, V^{*}=\frac{\theta S_{2}^{*}}{(\mu +\delta )},B^{*}=\frac{kI^{*}(\mu +\gamma +\sigma )}{\sigma (d+n\tau )}. \end{aligned}$$

$$\begin{aligned} S_{h}^{*}=\frac{A_{h}}{\beta _{h}(E^{*}+I^{*})+\mu _{h}}, I_{ah}^{*}=\frac{A_{h}-\mu _{h}S_{h}^{*}}{\mu _{h}+\delta _{h}+r}, \end{aligned}$$

here $$H=\beta +\beta \frac{\mu +\gamma }{\sigma }+\beta _{1}\frac{k(\mu +\gamma +\sigma )}{\sigma (d+n\tau )}$$.

#### Stability of equilibrium

**Theorem 2.** When $R_{0}<1,$ the disease-free equilibrium $E^{0}$ of model (2) is globally asymptotically stable, when $R_{0}>1,$ the disease-free equilibrium $E^{0}$ is unstable.

**Theorem 3.** When $R_{0}>1,$ the endemic equilibrium $E^{*}$ of model (2) is globally asymptotically stable.

**Proof.** The proof of Theorem 2 and 3 is given in A.2-A.4. of the supplementary material.

## Data calibration and numerical simulation

In this section, the numerical simulation to illustrate the theoretical results is performed firstly. Then, based on the incidence data of human brucellosis cases, model (2) is utilized to fit the data, and evaluate the accessibility of three widely applied strategies to control brucellosis.

### Verification of theoretical results

**Fig. 3:**
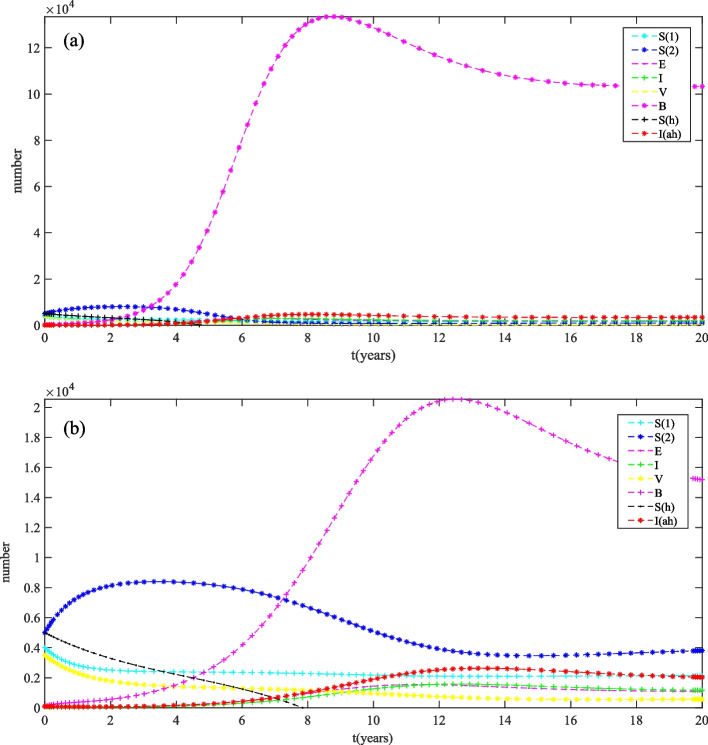
Stability diagrams for model (2) in $E^{0}$ (a) and $E^{*}$ (b)

Through the qualitative analysis of the model above, the global dynamics of model (2) is theoretically obtained. In order to describe the dynamic behavior of the model more vividly, firstly, the stability of the model is verified by numerical simulation. Taking initial value are $S_{1}(0)=4000, S_{2}(0)=5000, E(0)=7, I(0)=54, V(0)=3500, B(0)=100, S_{h}(0)=5000, I_{h}(0)=100,$ and $A=3000, \mu =0.25, \alpha =0.015, \gamma =0.15, \sigma =1, d=0.6, \tau =0.6, \varepsilon =0.4, \beta =0.000038, k=16, \beta _{1}=0.0000135, m=1.06, \theta =0.1, \delta =0.4, A_{h}=4000, \mu _{h}=0.3, \beta _{h}=0.000158, \delta _{h}=0.1, r=0.5, n=3.$ We can calculate that $R_{0}=0.66<1,$ it follows from Theorem 2 that disease-free equilibrium $E^{0}=(2406,6844,0,0,1360,0,1333,0)$ of model (2) is globally asymptotically stable, Fig. 3(a) supports this result. That is, brucellosis would disappear in the population and sheep, and the infected persons would tend to zero.

Next, we change  $n=1$  and the other parameters are the same as in Fig. 3(a), then we can obtain that $R_{0}=1.81>1$, and the condition of Theorem 3 is satisfied. The time series of the state variables are shown in Fig. 3(b). It follows from Theorem 3 that the endemic equilibrium point $E^*=(2163,401,1096,1203,817,15124,108,1985)$ is globally asymptotically stable, and the disease will persist.

### Fitting results of infected cases of human brucellosis in Ningxia

Ningxia is one of the regions with high prevalence of brucellosis in China. A total of 23,867 human cases of brucellosis were reported from 2005 to 2021. To perform the numerical simulations, we first need to estimate the model parameters. According to the existing literatures and related results of the Ningxia Statistical Yearbook, we estimated some parameters are listed in Table 1.

**Table 1:** Description of parameters in system (1)

Parameter	Interpretation	Value	Ref
*A*	The input munber of young sheep	100400	[30]
$\varepsilon$	Infection coefficient of individual lambs	3.9e-6	Estimated
$\beta$	The transmission rate from *I* to *S*	4.2e-14	Estimated
$\mu$	Individual natural mortality	0.22	[30]
$\sigma$	The incidence rate form *E* to *I*	3.4	[30]
$\gamma$	Slaughter rate of *I*	0.0023	Estimated
*k*	The brucella shedding rate by *I*	15	[40]
*d*	The natural decaying rate of *W*	3.577	[40]
*n*	Environmental disinfection times	1	[41]
$\tau$	The frequency of disinfection	1	[41]
*m*	Transfer rate from $S_{1}$ to $S_{2}$	0.4	[25]
$\theta$	Immunization rate in adult sheep	0.001	[41]
$\delta$	Loss of vaccination rate	0.4	[27]
$\alpha$	Sheep birth rate	0.305	Estimated
$\beta _{1}$	The transmission rate from *B* to $S_{h}$	7.15e-8	Estimated
$A_{h}$	The recruitment rate of human	10020	[30]
$\beta _{h}$	The transmission rate from *I* to $S_{h}$	3.18e-10	Estimated
$\mu _{h}$	The natural death rate of human	0.000495	[30]
$\delta _{h}$	The morbid death rate of humans	0.0001	[30]
*r*	The transfer rate from $I_{h}$ to $C_{h}$	0.64	[30]

Given that most human brucellosis cases are from rural areas and livestock and veterinary practitioners are likely to be exposed to infected sheep and contaminated environments, numerical simulations of rural populations in Ningxia in 2005 was used. It was calculated that $S_{h}(0)=1691922,$ $I_{h}(0)=32.$  According to Ningxia Statistical Yearbook from 2005 to 2021, by the end of 2004, there were about $N(0)= 505350000,$ $S_{1}+S_{2}=5017200.$ According to the stock of various sheep in Ningxia [42], $S_{1}(0)=1461700, S_{2}(0)=3368400.$ Since the vaccination rate for adult sheep was 0 in 2004, so we take $V(0)=0$. More detailed estimation process of the parameter values are as follows:

[*a*] The average annual recruitment of young sheep is 100,400, so $A=100400.$

[*b*] There are few prevention and control measures to eliminate sheep brucellosis before 2005, assuming that the vaccination rate of adult sheep is $\theta =0.01$ At the same time, farmers and herdsmen are not sterilized, assuming that the effective rate of disinfection is $\tau =1$ and the number of disinfection is $n=1.$

[*c*] Adult sheep life span is about 4-5 years, assuming that the individual mortality rate $\mu =1\div 4.5=0.22.$

[*d*] Since the incubation period ranges from 2 weeks to 7 months, it can not be accurately determined, it is assumed that the average time is 3.5 months and then the conversion rate of latent infection $\sigma =12\div 3.5=3.4.$

[*e*] Brucella shed rate to the environment $k=365\times 0.041=15,$ natural mortality rate $d=365\times 0.0098=3.577.$

[*f*] From Ningxia Statistical Yearbook, the natural mortality rate of the population is $\mu _h=0.000495,$ death rate due to disease is $\delta _h=0.001$, the average daily number of people reported is nominally 27, so $A_{h}=365\times 27=10020$.

**Fig. 4:**
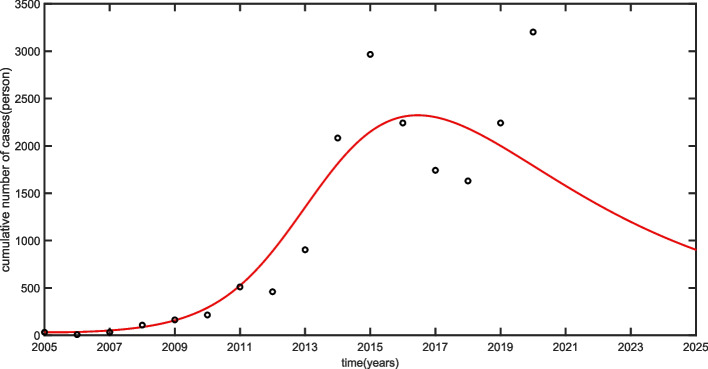
Fitting results of newly infected human brucellosis cases of Ningxia

The sum of $I_{ah}$ and $I_{ch}$ of model (1) is used to evaluate human brucellosis data in Ningxia from 2005 to 2020, and to predict the infection trend of human brucellosis. Due to the outbreak of COVID-19 in the spring of 2020, lockdown measures were implemented across the country, which may lead to a indirect effect on the trade activities and prolong indoor sheep feeding, resulting in the more frequent contact between humans and sheep, which may contribute to the high incidence of human brucellosis in 2020, thus, the fit data set excluding the human brucellosis data of 2020 in Ningxia. It is estimated that the basic reproductive number $R_{0}=1.47$ from 2005 to 2020, indicating that human brucellosis will persist in Ningxia under current control measures. Figure 4 shows that the fitting result is well alignment with the reported data of human brucellosis in Ningxia from 2005 to 2020.

## Accessibility evaluation result

To evaluate the accessibility of “Ningxia Brucellosis Prevention and Control Special Three-year Action Implementation Plan (2022-2024)”, we will consider the following three widely applied prevention and control strategies of brucellosis in Ningxia as follows:

1) slaughtering of sicked sheep;

2) increasing the health education;

3) increasing the immunization of adult sheep.

### The effect of slaughtering of sicked sheep on the control of brucellosis transmission

**Fig. 5:**
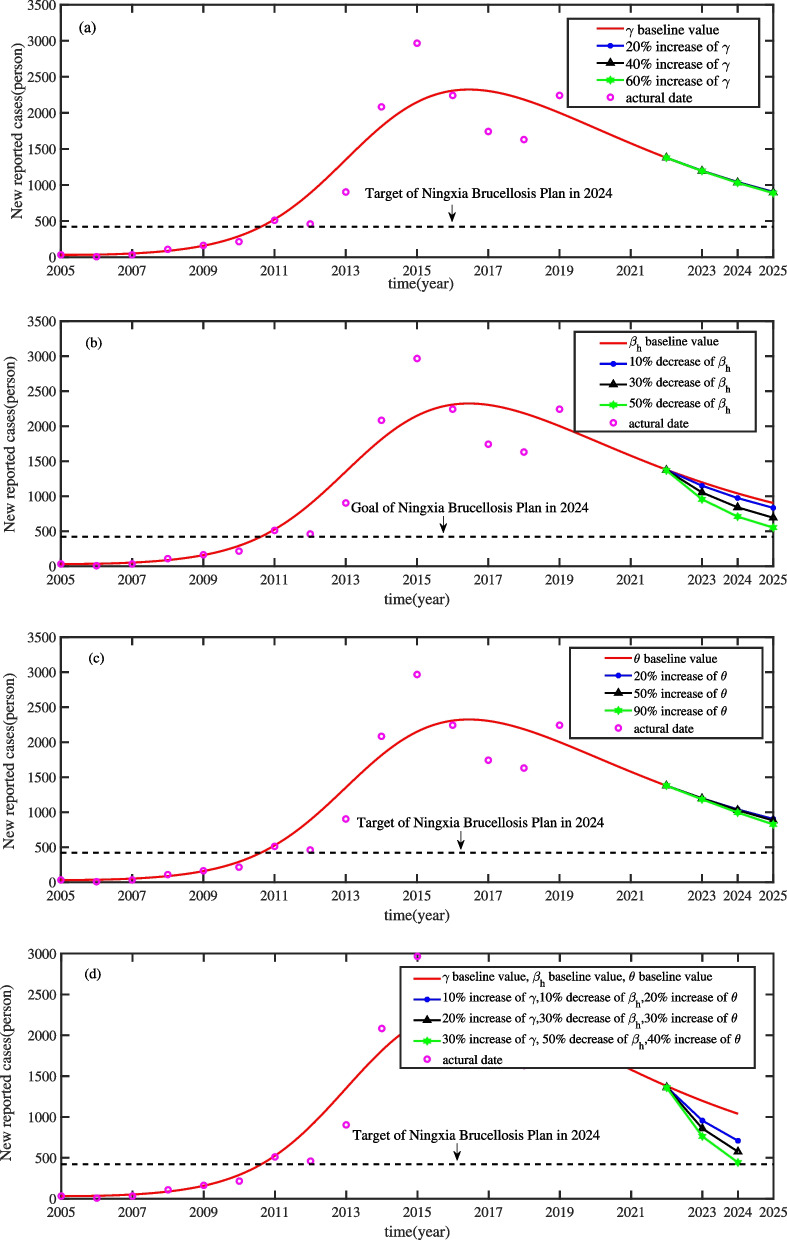
Influence of different control measures on brucellosis in Ningxia respectively. (a) Effects of different culling measures for sicked sheep, (b) The influences of strengthening publicity and education, (c) Effects of immunization the young sheep and (d) Effects of three comprehensive measures

In this subsection, we will discuss effect of slaughtering of sicked sheep ($\gamma$) on the control of human brucellosis in Ningxia. According to the above analysis, it is difficult to eliminate brucellosis in Ningxia in a short time. Fortunately, some measures can be implemented to reduce the prevalence of brucellosis, so that Ningxia can transform from a severe epidemic area to a general epidemic area. The effect of different slaughtering rates on the number of newly infected human brucellosis cases per year is shown in Fig. 5(a). The solid red line represents the baseline model. The solid blue line represents 10$\%$ increase in the slaughtering rate  $\gamma ,$ and the solid black line represents 50$\%$ increase in the slaughtering rate $\gamma .$ We can observe that it may not achieve the brucellosis control target in 2024 even if we will increase the slaughtering rate $\gamma$ by 90$\%$.

### The effect of health education on the control of brucellosis transmission

Based on the previous analysis, the brucellosis control is less effective if we only increase slaughtering rate of sicked sheep to a certain extent. Some other measures to control the spread of brucellosis should also be identified. The contact rate between susceptible and infected human brucellosis may be effectively reduce by health education. Thus, we will change the parameter ($\beta _{h}$) to discuss the effect of health education on the control of brucellosis transmission. As shown in Fig. 5(b), we can see that if we reduce $\beta _{h}$ by 10$\%$ or 30$\%$, the incidence of human brucellosis becomes lower, but the target of “Ningxia Brucellosis Prevention and Control Special Three-year Action Implementation Plan (2022-2024)” can not reach. Even if we reduce $\beta _{h}$ by 50$\%$, the target of the prevention and control plan may not be feasible. Thus, increasing health education to reduce the exposure to infected sheep and contaminated environments by wearing protective clothing and medical gloves and paying attention to hand hygiene may conducive to the control of brucellosis transmission, however, the control of human brucellosis in Ningxia still cannot be realized only relying on health education.

### The effect of immunization of adult sheep on the control of brucellosis transmission

Now, we are in a position to discuss the effect of immunization of adult sheep ($\theta$) on the control of brucellosis transmission. In numerical simulation, we will change the immunization rate in adult sheep $\theta$ to explore the effect of immunization of adult sheep on the control of brucellosis transmission. From the Fig. 5(c), we see that the higher immunization rate in adult sheep, the less incidence of human brucellosis, but the change is relatively slight. To be specific, when $\theta$ increases to 20$\%$, the number of human brucellosis cases is lower than that of the baseline model, and when $\theta$ increases to 90$\%$, the target of brucellosis control still not be achieved by 2024. These results show that the transmission of brucellosis could not be effectively controlled only by increasing the immunization rate of adult sheep.

### Mitigation of brucellosis transmission by the comprehensive control strategy

The above simulation results show that the single control strategy, even if scaled up to a very high level, is unlikely to achieve the the target of “Ningxia Brucellosis Prevention and Control Special Three-year Action Implementation Plan (2022-2024)” on schedule. Thus, a comprehensive control strategy, including slaughtering of sicked sheep, increasing the health education, and the immunization of adult sheep, should be considered to mitigate the brucellosis transmission in Ningxia. Figure 5(d) shows that increasing  $\gamma$ by 30$\%$, reducing $\beta _{h}$ to 50$\%$, and an increase of $\theta$ by 40$\%$, the prevention and control plan will be achieved in 2024. Therefore, we conclude that by using a single brucellosis control strategy may not meet the target of the prevention and control plan in 2024. To achieve the target, Ningxia should pay more attention to enhancing the comprehensive control strategy of brucellosis by combining the three widely applied prevention and control strategies.

## Discussion

According to the World Health Organization (WHO) situation report [43], about 500,000 cases of brucellosis are reported each year. However, brucellosis remains a neglected disease in many parts of the world. As a high prevalence area for brucellosis in China, the control work of brucellosis has gained some achievements in Ningxia recently [11]. Given the reemerging human brucellosis epidemic situation in Ningxia, the government of Ningxia has implemented the “Ningxia Brucellosis Prevention and Control Special Three-Year Action Implementation Plan (2022-2024)” to mitigate of brucellosis transmission. Thus, it is necessary to evaluate the accessibility of the three-year action plan for comprehensive prevention and control of brucellosis in Ningxia. According to the epidemiological history and transmission route of brucellosis, we proposed a coupled sheep-human-environment dynamic mathematical model to study the spread of brucellosis. First, we obtained the basic reproduction number $R_0$ of the model, which determined the dynamics: if $R_0<1$, the disease-free equilibrium E^0^of model is globally asymptotically stable, while $R_0<1$, the endemic equilibrium $E^*$ of model is globally asymptotically stable. Numerical simulation validated the corresponding theoretical result of Theorem 2 in Fig. 3.

The model was used to validate reported human brucellosis data from 2005-2020 in Ningxia by means of the Least square method, and it has good alignment with the reported human brucellosis data. We calculated that the basic reproduction number $R_{0}=1.47$, which suggested that human brucellosis in Ningxia will persist (as shown in Fig. 4). Furthermore, three widely applied control strategies of Brucellosis in Ningxia, that is, slaughtering of sicked sheep, health education to high risk practitioners, and immunization of adult sheep, are evaluated (see Fig. 5). The simulation results showed that a single brucellosis control strategy may not reach the goal of three-year action plan for comprehensive brucellosis prevention and control in 2024 and effective combining the three widely applied prevention and control strategies may be contribute to realize the target of Ningxia’s brucellosis control plan on time. In addition, these findings suggested that increasing  $\gamma$ by 30$\%$, reducing $\beta _{h}$ to 50$\%$, and an increase of $\theta$ by 40$\%$, the “Ningxia Brucellosis Prevention and Control Special Three-Year Action Implementation Plan (2022-2024)” will be achieved in 2024.

From a public health point of view, the health education to high risk practitioners is the most cost-effective measure to control brucellosis. In order to maximally reduce the loss of economic interests of breeding practitioner when the emergence of brucellosis, various forms of publicity and education (e.g., TV news, online platforms, posters, etc.) should be carried out to ensure that breeding practitioner have a more deep understanding of the prevention and control knowledge of brucellosis, and further convinced of the importance of immunization detection. In addition, veterinary authorities should continuously provide education and training programs to achieve practitioners’ awareness of prevention and transmission routes of brucellosis [44].

In addition, Fig. 5(c) shows that increasing the immunization rate of adult sheep may be benefit to the control of brucellosis. Interventions could be targeted toward specific groups, which would be particularly effective as an epidemic control measure [45]. For instance, immunization of adult sheep is one kind of methods to control brucellosis [21]. However, the current immunization rate of adult sheep is not enough to control the spread of brucellosis. To protect sheep suffered from the brucellosis, immunization should be carry out regularly during breeding period. Live vaccine strains of B. abortus RB51 and S19 are the most commonly used live vaccine strains for controlling brucellosis in sheep, which may enhance the immunity and resistance of sheep [46].

Regrading to the measure of slaughtering of sicked sheep, our result shows that increasing the slaughter rate of sicked sheep is not an effective measure to control of brucellosis in humans (see Fig. 5(a)). We increase the slaughter rate $\gamma$ from 10$\%$ to 50$\%$, but it still may not achieve the brucellosis control target in 2024 in Ningxia. We also observed that the effect of slaughtering of the sicked sheep is not as pronounced as that of health education. The positive test and slaughter policy is used to control brucellosis in countries or regions with low brucellosis infection [47]. However, this measure may not be a cost-effective approach in endemic areas from economic benefit viewpoint.

Nowadays, there is a ten year time-frame to achieve the goal of “Health China 2030”. The elimination of brucellosis by 2030 is an urgent task. Our accessibility assessment result suggests that effectively combining the three widely applied prevention and control strategies may reach the target of “Ningxia Brucellosis Prevention and Control Special Three-Year Action Implementation Plan (2022-2024)” In fact, the cornerstone of the epidemiology of zoonotic infectious diseases is the One Health concept. This disease will not be controlled or eradicated without collaboration between local and public partnerships. Thus, a combination of increasing of the animal immunization, positive testing and slaughtering, improving the breeding sanitary conditions and personal protection, and enhancing the health education of practitioner may be the optimal control strategy with a low cost [43]. Therefore, this still requires sustained improvement in brucellosis control to reduce the burden of brucellosis in Ningxia.

However, there are some limitations to this work. First, the mixed feeding between cattle and sheep in public farm is one of the most typical characteristics in brucellosis outbreak regions, which has a large influence on the spreading of brucellosis [24]. Due to sheep breeding industry account for 83.3$\%$ [41] of animal husbandry in Ningxia. We only consider the transmission route of sheep in modeling, however, the mixed cross infection should be discussed. Second, more reasonable mathematical modeling methods should be used to improve control strategies for targeted prevention and control of brucellosis. For instance, fractional derivative is also a good tools for describing substances and materials with memory and genetic properties. Recent studies have confirmed that fractional differential equations are more accurate in describing the development of things [48, 49]. Due to the complexity of brucellosis, fractional derivative modeling should be take into account to describe the memory and genetic properties in the transmission of brucellosis in future.

## Conclusion

In this paper, we propose a coupled sheep-human-environment dynamical mathematical model to evaluate the accessibility of “Ningxia Brucellosis Prevention and Control Special Three-Year Action Implementation Plan (2022-2024)”. The results indicate that the spread of brucellosis will persist in future and the current control target will not reach the goal by 2024. The evaluation of comprehensive control strategy of brucellosis suggest that increasing slaughtering rate $\gamma$ by 30$\%$, increasing health education to reduce infection rate of human $\beta _{h}$ to 50$\%$, and an increase of immunization rate of adult sheep $\theta$ by 40$\%$, the prevention and control plan will be achieved in 2024. These results can provide a reliable quantitative basis for further optimizing the prevention and control strategy of brucellosis in Ningxia.

## Supplementary Information

**Additional file 1.** Supplementary Material [39].

## Data Availability

The demographic data that support the findings of this study are available from Ningxia Statistical Yearbook (http://nxdata.com.cn/publish.htm?cn=G01) and the incidence data of brucellosis in Ningxia are available from Ningxia Center for Disease Control and Prevention (http://nxcdc.org/article/1940).
