# Supplementary material for: Accessibility of the three-year comprehensive prevention and control of brucellosis in Ningxia: a mathematical modeling study
Source: BMC Infect Dis. 2023 May 5;23:292. doi: 10.1186/s12879-023-08270-4 (PMC10161990; doi:10.1186/s12879-023-08270-4)
Supplement: Supplementary file 1 — Additional file 1. Supplementary Material [39]. [file 12879_2023_8270_MOESM1_ESM.pdf]

Supplementary Material

A.1. Proof of the positively invariant set

According to system (2)

$$\frac{\mathrm{d}S_1}{\mathrm{d}t}|_{S_1=0,S_2=0,V=0}=0,\ \frac{\mathrm{d}S_2}{\mathrm{d}t}|_{S_1=0,S_2=0,V=0}=0,\ \frac{\mathrm{d}E}{\mathrm{d}t}|_{S_1=0,S_2=0,E=0}=0,$$

$$\frac{\mathrm{d}I}{\mathrm{d}t}|_{E=0,I=0}=0,\ \frac{\mathrm{d}V}{\mathrm{d}t}|_{S_2=0,V=0}=0,\ \frac{\mathrm{d}B}{\mathrm{d}t}|_{E=0,I=0,B=0}=0,\ \frac{\mathrm{d}S_h}{\mathrm{d}t}|_{S_h=0}=0,\ \frac{\mathrm{d}I_{ah}}{\mathrm{d}t}|_{S_h=0,I_{ah}=0}=0,$$

all of the above values are non-negative in the bounded plane of $R_8^+=\{(x_0,x_1,x_2,x_3,x_4,x_5,x_6,x_7):x_i\geq 0, i=0,1,2,3,4,5,6,7\}$. If the initial solution of system (2) is in R_8^+, then all the solutions are non-negative. Let $(S_1,S_2,E,I,V,B,S_h,I_{ah})$ be the solution under the initial condition, satisfy $N=S_1+S_2+E+I+V, N_h=S_h+I_{ah}$, then

$$\frac{\mathrm{d}N}{\mathrm{d}t}=A+\alpha(S_2+V)-\mu N-\gamma I\leq A-(\mu-\alpha)N.$$

It follows that

$$\lim_{t\to\infty}\sup N(t)=\frac{A}{\mu-\alpha}.$$

From system (2), one can obtain that

$$\frac{\mathrm{d}B}{\mathrm{d}t}\leq kN-(d+n\tau)B\leq\frac{kA}{\mu-\alpha}-(d+n\tau)B,$$

then

$$\lim_{t\to\infty}\sup B(t)=\frac{kA}{(\mu-\alpha)(d+n\tau)}.$$

Similarly, it can be obtained

$$\lim_{t\to\infty}\sup N_h=\frac{A_h}{\mu_h}.$$

This completes the proof.

A.2. Proof of the local stability of E^0

The Jacobian matrix of system (2) at E^0 is given by

$$J(E^0)=\begin{pmatrix}\Omega_1 & 0 & 0\\ \Omega_2 & \Omega_3 & 0\\ \Omega_4 & 0 & \Omega_5\end{pmatrix}$$

where

$$F=\left(\begin{array}{ccc}\varepsilon\beta S_1^0+\beta S_2^0 & \varepsilon\beta S_1^0+\beta S_2^0 & \varepsilon\beta_1 S_1^0+\beta_1 S_2^0\\ 0 & 0 & 0\\ 0 & 0 & 0\end{array}\right), V=\left(\begin{array}{ccc}\mu+\sigma & 0 & 0\\ -\sigma & \mu+\gamma & 0\\ -k & -k & d+n\tau\end{array}\right),$$

Let $\Omega_1=F-V$

$$\Omega_1=\left(\begin{array}{ccc}\varepsilon\beta S_1^0+\beta S_2^0-(\mu+\sigma) & \varepsilon\beta S_1^0+\beta S_2^0 & \varepsilon\beta_1 S_1^0+\beta_1 S_2^0\\ \sigma & -(\mu+\gamma) & 0\\ k & k & -(d+n\tau)\end{array}\right),$$

$$\Omega_2=\left(\begin{array}{ccc}-\varepsilon\beta S_1^0 & -\varepsilon\beta S_1^0 & -\varepsilon\beta S_1^0\\ -\beta_2 S_2^0 & -\beta_2 S_2^0 & -\beta_1 S_2^0\\ 0 & 0 & 0\end{array}\right), \Omega_3=\left(\begin{array}{ccc}-(\mu+m) & \alpha & \alpha\\ m & -(\mu+\theta) & \delta\\ 0 & \theta & -(\mu+\delta)\end{array}\right),$$

$$\Omega_4=\left(\begin{array}{ccc}-\beta_h S_h^0 & -\beta_h S_h^0 & 0\\ 0 & 0 & 0\end{array}\right), \Omega_5=\left(\begin{array}{cc}-\mu_h & 0\\ 0 & -(\mu_h+\delta_h+r)\end{array}\right).$$

Define $s(\Omega_1)$ be the spectral bound of matrix Ω_1. $s(\Omega_1)=\max\{Re\lambda : \lambda \text{ is an eigenvalue of } \Omega_1\}$, according to Theorem 2 of [?], it can be obtained

$$R_0>1\Leftrightarrow s(\Omega_1)>0, R_0<1\Leftrightarrow s(\Omega_1)<0.$$

The characteristic equation of $J(\Omega_3)$ is

$$p(\lambda)=\lambda^3+b_1\lambda^2+b_2\lambda+b_3=0,$$

where

$$\begin{aligned}b_1&=3\mu+\theta+m+\delta,\\ b_2&=\mu(\mu+\theta+\delta)+(\mu+m)(2\mu+\theta+\delta)-m\alpha,\\ b_3&=\mu(\mu+m)(\mu+\theta+\delta)-m\alpha(\mu+\theta+\delta).\end{aligned}$$

Obviously, $b_1>0, b_2>0, b_3>0$, and

$$\begin{aligned}b_1b_2-b_3=&(3\mu+\theta+\delta+m)[(\mu+\theta+\delta)(2\mu+m)+\mu(\mu+m)-m\alpha]\\ &-(\mu+\theta+\delta)(\mu^2+m\mu-m\alpha)\\ >&(3\mu+\theta+\delta+m)(\mu+\theta+\delta)(2\mu+m)-(\mu+\theta+\delta)(\mu^2+m\mu)\\ &+(\mu+\theta+\delta)m\alpha\\ >&0.\end{aligned}$$

According to the Routh-Hurwitz criterion, the matrix $J(\Omega_3)$ has eigenvalues with negative real parts. Therefore, when $R_0<1$, there are $s(\Omega_1)<0$ and $s(J|E^0)<0$. Thus, the disease-free equilibrium E^0 of system (2) is locally asymptotically stable. When $R_0>1$, there is a positive eigenvalue, the disease-free equilibrium E^0 is unstable.

A.3. Proof of the global stability of E^0

First, we define the Lyapunov function as follows

$$
\begin{aligned}
L_1(t) = {} & S_1(t) - S_1^0 - S_1^0 \ln\frac{S_1(t)}{S_1^0} + S_2(t) - S_2^0 - S_2^0 \ln\frac{S_2(t)}{S_2^0} + V(t) - V^0 - V^0 \ln\frac{V(t)}{V^0} \\
& + E + \frac{(\mu+\sigma)(d+n\tau) - (d+n\tau)(\varepsilon\beta S_1^0 + \beta S_2^0) - k(\varepsilon\beta_1 S_1^0 + \beta_1 S_2^0)}{\sigma(d+n\tau)} I \\
& + \frac{\varepsilon\beta_1 S_1^0 + \beta_1 S_2^0}{d+n\tau} B.
\end{aligned}
$$

From equation (4), when $R_0 < 1$, it can be obtained

$$
(\mu+\sigma)(d+n\tau) > (d+n\tau)(\varepsilon\beta S_1^0 + \beta S_2^0) + k(\varepsilon\beta_1 S_1^0 + \beta_1 S_2^0),
$$

$$
\frac{(\mu+\sigma)(d+n\tau) - (d+n\tau)(\varepsilon\beta S_1^0 + \beta S_2^0) - k(\varepsilon\beta_1 S_1^0 + \beta_1 S_2^0)}{\sigma(d+n\tau)} > 0,
$$

Taking derivative along with the solution of model (2), we have

$$
\begin{aligned}
\frac{\mathrm{d}L_1(t)}{\mathrm{d}t} = {} & (1 - \frac{S_1^0}{S_1})[A + \alpha(S_2+V) - \varepsilon(\beta S_1 I + \beta S_1 E + \beta_1 S_1 B) \\
& - \frac{A + \alpha(S_2^0 + V^0)}{S_1^0} S_1] + (1 - \frac{S_2^0}{S_2})[m S_1 + \delta V - (\beta S_2 I + \beta S_2 E \\
& + \beta_1 S_2 B) - \frac{m S_1^0 + \delta V^0}{S_2^0} S_2] + (1 - \frac{V^0}{V})[\theta S_2 - \frac{\theta S_2^0}{V^0} V] \\
& + \varepsilon(\beta S_1 I + \beta S_1 E + \beta_1 S_1 B) + (\beta S_2 I + \beta S_2 E + \beta_1 S_2 B) - (\mu+\sigma)E \\
& + \frac{(\mu+\sigma)(d+n\tau) - (d+n\tau)(\varepsilon\beta S_1^0 + \beta S_2^0) - k(\varepsilon\beta_1 S_1^0 + \beta_1 S_2^0)}{\sigma(d+n\tau)} \\
& [\sigma E - (\mu+\gamma) I] + \frac{\varepsilon\beta_1 S_1^0 + \beta_1 S_2^0}{d+n\tau}[k(E+I) - (d+n\tau)B],
\end{aligned}
\tag{A1}
$$

where $A = \mu S_1^0 + \mu V^0 + \mu S_2^0 - \alpha(S_2^0 + V^0), m S_1^0 = \mu V^0 + \mu S_2^0, \theta S_2^0 = (\mu+\delta)V^0,$ substituting into (A1), then

$$
\begin{aligned}
\frac{\mathrm{d}L_1(t)}{\mathrm{d}t} = {} & \mu S_1^0 (2 - \frac{S_1^0}{S_1} - \frac{S_1}{S_1^0}) + (\mu V^0 - \alpha V^0)(4 - \frac{S_1^0}{S_1} - \frac{S_2^0 S_1}{S_1^0 S_2} - \frac{V}{V^0} - \frac{S_2 V^0}{S_2^0 V}) \\
& + (\mu S_2^0 - \alpha S_2^0)(3 - \frac{S_1^0}{S_1} - \frac{S_2}{S_2^0} - \frac{S_2^0 S_1}{S_1^0 S_2}) + \delta V^0 (2 - \frac{S_2^0 V}{S_2 V^0} - \frac{S_2 V^0}{S_2^0 V}) \\
& + \alpha S_2^0 (2 - \frac{S_2^0 S_1}{S_1^0 S_2} - \frac{S_1^0 S_2}{S_2^0 S_1}) + \alpha V^0 (3 - \frac{S_2 V^0}{S_2^0 V} - \frac{S_1^0 V}{S_1 V^0} - \frac{S_2^0 S_1}{S_1^0 S_2}) \\
& + \frac{(\mu+\sigma)(\mu+\gamma)}{\sigma} I(R_0 - 1).
\end{aligned}
$$

Therefore, when $R_0 < 1$, $\frac{\mathrm{d}L_1(t)}{\mathrm{d}t} < 0$ and $\frac{\mathrm{d}L_1(t)}{\mathrm{d}t} = 0$ if and only if $S_1 = S_1^0, S_2 = S_2^0, E = 0, I = 0, V = V^0, B = 0$.

In order to prove the global stability of the disease-free equilibrium at E^0, consider

system (2) with the remaining two equations in disease-free state,

$$\begin{cases} \dfrac{\mathrm{d}S_h}{\mathrm{d}t} = A_h - \mu_h S_h, \\ \dfrac{\mathrm{d}I_{ah}}{\mathrm{d}t} = -(\mu_h + \delta_h + r) I_{ah}. \end{cases}$$

Constructing the Lyapunov function as follows

$$L_2(t) = (S_h - S_h^0 - S_h^0 \ln \frac{S_h}{S_h^0}) + I_{ah}.$$

Taking the derivative at the disease-free equilibrium point $E_h^0 = (S_h^0, I_{ah}^0)$ of system (2), we can get

$$\begin{aligned} \frac{\mathrm{d}L_2(t)}{\mathrm{d}t} &= \frac{S_h - S_h^0}{S_h}(A_h - \mu_h S_h) - (\mu_h + \delta_h + r) I_{ah} \\ &= -\frac{A_h}{S_h}(S_h - S_h^0)^2 - (\mu_h + \delta_h + r) I_{ah}. \end{aligned}$$

It can be obtained $\frac{\mathrm{d}L_2(t)}{\mathrm{d}t} \leq 0$, and $\frac{\mathrm{d}L_2(t)}{\mathrm{d}t} = 0$ if and only if $S_h = S_h^0, I_{ah} = I_{ah}^0 = 0$. From the $LaSalle's$ invariant principle, the disease-free equilibrium point $E_h^0 = (S_h^0, I_{ah}^0)$ is globally asymptotically stable within Γ.

In summary, when $R_0 < 1$, the disease-free equilibrium point E^0 of system (2) is globally asymptotically stable.

A.4. Proof of the global stability of E^*

The Jacobian matrix $J(E^*)$ at the endemic equilibrium E^* is

$$J(E^*) = \begin{pmatrix} \Psi_1 & \Psi_2 & 0 \\ \Psi_3 & \Psi_4 & 0 \\ \Psi_5 & \Psi_6 & \Psi_7 \end{pmatrix}$$

where

$$\Psi_1 = \begin{pmatrix} -\mu - m - \varepsilon(\beta I + \beta E + \beta_1 B) & \alpha & -\varepsilon\beta S_1 \\ m & -\theta - \mu - \beta(I + E) - \beta_1 B & -\beta S_2 \\ \varepsilon(\beta I + \beta E + \beta_1 B) & \beta(I + E) + \beta_1 B & -(\mu + \sigma) \end{pmatrix},$$

$$\Psi_2 = \begin{pmatrix} -\varepsilon\beta S_1 & \alpha & -\varepsilon\beta_1 S_1 \\ -\beta S_2 & \delta & -\beta_1 S_2 \\ \varepsilon\beta S_1 + \beta S_2 & 0 & \varepsilon\beta_1 S_1 + \beta_1 S_2 \end{pmatrix}, \Psi_3 = \begin{pmatrix} 0 & 0 & \sigma \\ 0 & \theta & 0 \\ 0 & 0 & k \end{pmatrix},$$

$$\Psi_4 = \begin{pmatrix} -(\mu + \gamma) & 0 & 0 \\ 0 & -(\mu + \sigma) & 0 \\ k & 0 & -(d + n\tau) \end{pmatrix}, \Psi_5 = \begin{pmatrix} 0 & 0 & -\beta_h S_h \\ 0 & 0 & \beta_h S_h \end{pmatrix},$$

$$\Psi_6 = \begin{pmatrix} -\beta_h S_h & 0 & 0 \\ \beta_h S_h & 0 & 0 \end{pmatrix}, \Psi_7 = \begin{pmatrix} -\beta_h(E + I) - \mu_h & 0 \\ \beta_h(E + I) & -(\mu_h + \delta_h + r) \end{pmatrix},$$

The characteristic equation of $J(\Psi_1)$ is

$$\begin{aligned}|xI - J(\Psi_1)| &= (x+\mu+m+\varepsilon\beta I+\varepsilon\beta E+\varepsilon\beta_2 B)[x^2+x(\beta^2S_2I+\beta^2S_2E+\beta\beta_1S_2B \\ &\quad -\varepsilon^2S_1I\beta^2-\varepsilon^2S_1E\beta^2-\varepsilon^2\beta\beta_1S_1B-m\alpha)+(-\varepsilon^2S_1I\beta^2-\varepsilon^2S_1E\beta^2 \\ &\quad -\varepsilon^2\beta\beta_1S_1B)(\theta+\mu+\beta I+\beta E+\beta_1B)+(\beta^2S_2I+\beta^2S_2E+\beta\beta_1S_2B) \\ &\quad (\mu+m+\varepsilon\beta I+\varepsilon\beta E+\varepsilon\beta B)+(\mu+\sigma)m\alpha]+(x+\theta+\mu+\beta I+\beta E \\ &\quad +\beta_1B)(x+\mu+\sigma) \\ &= x^3+a_2x^2+a_1x+a_0\end{aligned}$$

where

$$a_2 = \mu+m+\varepsilon\beta I+\varepsilon\beta E+\varepsilon\beta_2B,$$

$$\begin{aligned}a_1 &= \beta^2S_2I+\beta^2S_2E+\beta\beta_1S_2B-\varepsilon^2S_1I\beta^2-\varepsilon^2S_1E\beta^2-\varepsilon^2\beta\beta_1S_1B-m\alpha \\ &\quad +\mu+\sigma+\theta+\mu+\beta I+\beta E+\beta_1B\end{aligned}$$

$$\begin{aligned}a_0 &= (\beta^2S_2I+\beta^2S_2E+\beta\beta_1S_2B)(\mu+m+\varepsilon\beta I+\varepsilon\beta E+\varepsilon\beta B) \\ &\quad -\varepsilon^2S_1(I\beta^2+E\beta^2+\beta\beta_1B)(\theta+\mu+\beta I+\beta E+\beta_1B) \\ &\quad +(\mu+\sigma)m\alpha+(\theta+\mu+\beta I+\beta E+\beta_1B)(\mu+\sigma)\end{aligned}$$

Obviously, $a_0 > 0, a_1 > 0, a_2 > 0$, and

$$\begin{aligned}a_1a_2-a_0 &= (\theta+\mu+\beta I+\beta E+\beta_1B)(\varepsilon^2S_1I\beta^2+\varepsilon^2S_1E\beta^2+\varepsilon^2\beta\beta_1S_1B+\mu+\sigma) \\ &\quad +(\mu+m+\varepsilon\beta I+\varepsilon\beta E+\varepsilon\beta B)(2\beta^2S_2I+2\beta^2S_2E+2\beta\beta_1S_2B-\varepsilon^2S_1I\beta^2 \\ &\quad +\varepsilon^2S_1E\beta^2+\varepsilon^2\beta\beta_1S_1B-m\alpha+2\mu+\sigma+\theta+\beta I+\beta E+\beta_1B) \\ &> 0.\end{aligned}$$

According to the Routh-Hurwitz criterion,the matrix $J(\Psi_1)$ has eigenvalues with negative real parts. Thus, the endemic equilibrium E^* of system (2) is locally asymptotically stable.

Next, we define the Lyapunov function as follows

$$\begin{aligned}L_3(t) &= S_1(t)-S_1^*-S_1^*\ln\frac{S_1(t)}{S_1^*}+S_2(t)-S_2^*-S_2^*\ln\frac{S_2(t)}{S_2^*} \\ &\quad +V(t)-V^*-V^*\ln\frac{V(t)}{V^*}+E(t)-E^*-E^*\ln\frac{E(t)}{E^*} \\ &\quad +(\frac{\varepsilon\beta S_1^*I^*}{\sigma E^*}+\frac{kI^*(\varepsilon\beta_1S_1^*B^*+\beta_1S_2^*B^*)}{\sigma E^*(kE^*+kI^*)})[I(t)-I^*-I^*\ln\frac{I(t)}{I^*}] \\ &\quad +\frac{\varepsilon\beta_1S_1^*B^*+\beta_1S_2^*B^*}{kE^*+kI^*}[B(t)-B^*-B^*\ln\frac{B(t)}{B^*}]\end{aligned}$$

Taking derivative along with the solution of model (2), we have

$$
\begin{aligned}
\frac{dL_3(t)}{dt} &= (1-\frac{S_1^*}{S_1})[A+\alpha(S_2+V)-\varepsilon(\beta S_1 I+\beta S_1 E+\beta_1 S_1 B)] \\
&- (1-\frac{S_1^*}{S_1})[\frac{A+\alpha(S_2^*+V^*)-\varepsilon(\beta S_1^* I^*+\beta S_1^* E+\beta_1 S_1^* B^*)}{S_1^*}S_1] \\
&+ (1-\frac{S_2^*}{S_2})[mS_1+\delta V-(\beta S_2 I+\beta S_2 E+\beta_1 S_2 B))] \\
&- (1-\frac{S_2^*}{S_2})[\frac{mS_1^*+\delta V^*-(\beta S_2^* I^*+\beta S_2^* E^*+\beta_1 S_2^* B^*)}{S_2^*}S_2] \\
&+ (1-\frac{E^*}{E})[\varepsilon(\beta S_1 I+\beta S_1 E+\beta_1 S_1 B)+\beta S_2 I+\beta S_2 E+\beta_1 S_2 B)] \\
&- (1-\frac{E^*}{E})[\frac{\varepsilon(\beta S_1^* I^*+\beta S_1^* E^*+\beta_1 S_1^* B^*)+\beta S_2^* I^*+\beta S_2^* E^*+\beta_1 S_2^* B^*)}{E^*}E] \\
&+ (1-\frac{V^*}{V})[\theta S_2-\frac{\theta S_2^*}{V^*}V]+(\varepsilon\beta S_1^* I^*+\beta S_2^* I^*)(1-\frac{I^*}{I})(\frac{E}{E^*}-\frac{I}{I^*}) \\
&+ \frac{kI^*(\varepsilon\beta_1 S_1^* B^*+\beta_1 S_2^* B^*)}{kE^*+kI^*}(1-\frac{I^*}{I})(\frac{E}{E^*}-\frac{I}{I^*}) \\
&+ \frac{\varepsilon\beta_1 S_1^* B^*+\beta_1 S_2^* B^*}{kE^*+kI^*}kE^*(1-\frac{B^*}{B})(\frac{E}{E^*}-\frac{B}{B^*}) \\
&+ \frac{\varepsilon\beta_1 S_1^* B^*+\beta_1 S_2^* B^*}{kE^*+kI^*}kI^*(1-\frac{B^*}{B})(\frac{I}{I^*}-\frac{B}{B^*})
\end{aligned}
$$

Taking note that

$$\theta S_2^*=(\mu+\delta)V^*, mS_1^*=\mu(S_2^*+V^*)-\delta V^*+\beta S_2^* I^*+\beta S_2^* E^*+\beta_1 S_2^* B^*$$

$A=\mu(S_1^*+S_2^*+V^*)-\alpha(S_2^*+V^*)+\varepsilon(\beta S_1^* I^*+\beta, S_1^* E^*+\beta_1 S_1^* B^*)+\beta S_2^* I^*+\beta S_2^* E^*+\beta_1 S_2^* B^*.$

Then

$$\begin{aligned}
\frac{dL_3(t)}{dt} &= \mu S_1^*(2-\frac{S_1^*}{S_1}-\frac{S_1}{S_1^*})+\mu V^*(4-\frac{S_1^*}{S_1}-\frac{S_2^*S_1}{S_1^*S_2}-\frac{V}{V^*}-\frac{S_2V^*}{S_2^*V})\\
&+\mu S_2^*(3-\frac{S_1^*}{S_1}-\frac{S_2}{S_2^*}-\frac{S_2^*S_1}{S_1^*S_2})+\delta V^*(2-\frac{S_2^*V}{S_2V^*}-\frac{S_2V^*}{S_2^*V})\\
&-\alpha S_2^*(1-\frac{S_1^*}{S_1}-\frac{S_2}{S_2^*}+\frac{S_2S_1^*}{S_1S_2^*})-\alpha V^*(1-\frac{S_1^*}{S_1}-\frac{V}{V^*}+\frac{S_1^*V}{S_1V^*})\\
&+\varepsilon\beta S_1^*I^*(3-\frac{S_1^*}{S_1}-\frac{I^*E}{IE^*}-\frac{E^*S_1I}{ES_1^*I^*})+\varepsilon\beta S_1^*E^*(2-\frac{S_1^*}{S_1}-\frac{S_1}{S_1^*})\\
&+\varepsilon\beta_1 S_1^*B^*(2-\frac{S_1^*}{S_1}-\frac{E}{E^*}+\frac{B}{B^*}-\frac{E^*S_1B}{ES_1^*B^*})+\beta S_2^*E^*(3-\frac{S_1^*}{S_1}-\frac{S_1S_2^*}{S_2S_1^*}-\frac{S_2}{S_2^*})\\
&+\beta S_2^*I^*(4-\frac{S_1^*}{S_1}-\frac{I^*E}{IE^*}-\frac{S_1S_2^*}{S_2S_1^*}-\frac{E^*S_2I}{ES_2^*I^*})\\
&+\beta_1 S_2^*B^*(3-\frac{S_1^*}{S_1}-\frac{S_1S_2^*}{S_2S_1^*}-\frac{E}{E^*}+\frac{B}{B^*}-\frac{E^*S_2B}{ES_2^*B^*})\\
&+\frac{kI^*\varepsilon\beta_1S_1^*B^*}{kE^*+kI^*}(2+\frac{E}{E^*}-\frac{B}{B^*}-\frac{I^*E}{IE^*}-\frac{B^*I}{BI^*})\\
&+\frac{kI^*\beta_1S_2^*B^*}{kE^*+kI^*}(2+\frac{E}{E^*}-\frac{B}{B^*}-\frac{I^*E}{IE^*}-\frac{B^*I}{BI^*})\\
&+\frac{kE^*\varepsilon\beta_1S_1^*B^*}{kE^*+kI^*}(\frac{E}{E^*}-\frac{B}{B^*}-\frac{B^*E}{BE^*}+1)+\frac{kE^*\beta_1S_2^*B^*}{kE^*+kI^*}(\frac{E}{E^*}-\frac{B}{B^*}-\frac{B^*E}{BE^*}+1)\\
&= \mu S_1^*(2-\frac{S_1^*}{S_1}-\frac{S_1}{S_1^*})+(\mu V^*-\alpha V^*)(4-\frac{S_1^*}{S_1}-\frac{S_2^*S_1}{S_1^*S_2}-\frac{V}{V^*}-\frac{S_2V^*}{S_2^*V})\\
&+(\mu S_2^*-\alpha S_2^*)(3-\frac{S_1^*}{S_1}-\frac{S_2}{S_2^*}-\frac{S_2^*S_1}{S_1^*S_2})+\delta V^*(2-\frac{S_2^*V}{S_2V^*}-\frac{S_2V^*}{S_2^*V})\\
&+\alpha S_2^*(2-\frac{S_2S_1^*}{S_1S_2^*}-\frac{S_2^*S_1}{S_1^*S_2})+\alpha V^*(3-\frac{S_2^*S_1}{S_1^*S_2}-\frac{S_1^*V}{V^*S_1}-\frac{S_2V^*}{S_2^*V})\\
&+\varepsilon\beta S_1^*I^*(3-\frac{S_1^*}{S_1}-\frac{I^*E}{IE^*}-\frac{E^*S_1I}{ES_1^*I^*})+\varepsilon\beta S_1^*E^*(2-\frac{S_1^*}{S_1}-\frac{S_1}{S_1^*})\\
&+\beta S_2^*I^*(4-\frac{S_1^*}{S_1}-\frac{I^*E}{IE^*}-\frac{S_1S_2^*}{S_2S_1^*}-\frac{E^*S_2I}{ES_2^*I^*})+\beta S_2^*E^*(3-\frac{S_1^*}{S_1}-\frac{S_1S_2^*}{S_2S_1^*}-\frac{S_2}{S_2^*})\\
&+\frac{kI^*\varepsilon\beta_1S_1^*B^*}{kE^*+kI^*}(4-\frac{S_1^*}{S_1}-\frac{I^*E}{IE^*}-\frac{B^*I}{BI^*}-\frac{E^*S_1B}{ES_1^*B^*})\\
&+\frac{kI^*\beta_1S_2^*B^*}{kE^*+kI^*}(5-\frac{S_1^*}{S_1}-\frac{S_1S_2^*}{S_2S_1^*}-\frac{I^*E}{IE^*}-\frac{B^*I}{BI^*}-\frac{E^*S_2B}{ES_2^*B^*})\\
&+\frac{kE^*\varepsilon\beta_1S_1^*B^*}{kE^*+kI^*}(3-\frac{S_1^*}{S_1}-\frac{B^*E}{BE^*}-\frac{E^*S_1B}{ES_1^*B^*})\\
&+\frac{kE^*\beta_1S_2^*B^*}{kE^*+kI^*}(4-\frac{S_1^*}{S_1}-\frac{S_1S_2^*}{S_2S_1^*}-\frac{B^*E}{BE^*}-\frac{E^*S_2B}{ES_2^*B^*}).
\end{aligned}$$

Since

$$2-\frac{S_1^*}{S_1}-\frac{S_1}{S_1^*}\le 0, 3-\frac{S_1^*}{S_1}-\frac{I^*E}{IE^*}-\frac{E^*S_1I}{ES_1^*I^*}\le 0$$

$$4-\frac{S_1^*}{S_1}-\frac{I^*E}{IE^*}-\frac{S_1S_2^*}{S_2S_1^*}-\frac{E^*S_2I}{ES_2^*I^*}\le 0, 5-\frac{S_1^*}{S_1}-\frac{S_1S_2^*}{S_2S_1^*}-\frac{I^*E}{IE^*}-\frac{B^*I}{BI^*}-\frac{E^*S_2B}{ES_2^*B^*}\le 0.$$

Therefore $\frac{dL_3(t)}{dt} \leq 0$, $\frac{dL_3(t)}{dt} = 0$ if and only if $S_1 = S_1^*, S_2 = S_2^*, E = E^*, I = I^*, V = V^*, B = B^*$. In order to prove the global stability of the endemic equilibrium E^*, we need to consider the systems of the remaining two equations of system (2) in the endemic state

$$\begin{cases} \dfrac{\mathrm{d}S_h}{\mathrm{d}t} = A_h - \beta_h S_h(E^* + I^*) - \mu_h S_h, \\ \dfrac{\mathrm{d}I_{ah}}{\mathrm{d}t} = \beta_h S_h(E^* + I^*) - (\mu_h + \delta_h + r)I_{ah}. \end{cases}$$

We define the Lyapunov function as follows

$$L_4(t) = S_h - S_h^* - S_h^* \ln \frac{S_h}{S_h^*} + I_{ah} - I_{ah}^* - I_{ah}^* \ln \frac{I_{ah}}{I_{ah}^*},$$

Taking the derivative at the endemic equilibrium point E^* of system (2), we can get

$$\begin{aligned} \frac{dL_4(t)}{dt} &= (1 - \frac{S_h^*}{S_h})[\beta_h S_h^*(E + I) + \mu_h S_h^* - \beta_h S_h(E^* + I^*) - \mu_h S_h] \\ &\quad + (1 - \frac{I_{ah}^*}{I_{ah}})[\beta_h S_h(E^* + I^*) - (\mu_h + \delta_h + r)I_{ah}] \\ &= -\frac{\beta_h(E^* + I^*) + \mu_h}{S_h}(S_h - S_h^*)^2 - \frac{\mu_h + \delta_h + r}{I_{ah}}(I_{ah} - I_{ah}^*)^2. \end{aligned}$$

Therefore $\frac{dL_4(t)}{dt} \leq 0, \frac{dL_4(t)}{dt} = 0$ if and only if $S_h = S_h^*, I_{ah} = I_{ah}^*$. From the $LaSalle's$ invariant principle, the endemic equilibrium point E^* is globally asymptotically stable within Γ.
